# Energy Harvesting Combat Boot for Satellite Positioning

**DOI:** 10.3390/mi9050244

**Published:** 2018-05-17

**Authors:** Haluk Akay, Ruize Xu, Dexter Chew Xuan Han, T. Hui Teo, Sang-Gook Kim

**Affiliations:** 1 Massachusetts Institute of Technology, 77 Massachusetts Ave., 1-306, Cambridge, MA 02139, USA; haluk@mit.edu (H.A.); xuruize@gmail.com (R.X.); 2 Singapore University of Technology and Design, 8 Somapah Rd., Singapore 487372, Singapore; dexter_chew@mymail.sutd.edu.sg (D.C.X.H.); tthui@sutd.edu.sg (T.H.T.)

**Keywords:** energy harvesting, GPS-equipped footwear, micro turbines, foot-step driven airflow

## Abstract

Most portable electronic devices are power-limited by battery capacity, and recharging these batteries often interrupts the user’s experience with the device. The product presented in this paper provides an alternative to powering portables by converting regular human walking motion to electricity. The device harvests electric power using air bulbs, distributed in the sole of a shoe to drive a series of micro-turbines connected to small DC motors. The number and position of air bulbs is optimized to harvest the maximum airflow from each foot-strike. The system is designed to continuously drive the micro-turbines by utilizing both outflow and inflow from the air bulbs. A prototype combat boot was fitted on the right foot of a 75 kg test subject, and produced an average continuous power on the order of 10 s of mW over a 22 Ω load during walking at 3.0 mph. This combat boot provides enough electric power to a passive GPS tracker that periodically relays geographical coordinates to a smartphone via satellite without battery replacement.

## 1. Introduction

The human body maintains a diverse portfolio of physical activity. Certain human processes, such as the blood coursing through veins and arteries, run continuously in the background, while others, such as sprinting, occur as acutely executed functions of motion. Regardless of the nature of each activity, every physical motion of the human body presents an opportunity to harvest energy and power a portable device without relying on a battery in remote areas [1]. Of these activities, the impact of the human foot on the ground during walking and running presents the greatest occasion to harvest energy in terms of energy expenditure [2]. While bipedal movement is not a continuous activity, its occurrence is correlated with the need for electric power. This raises the challenge of converting foot-falls into usable electric power.

Regular walking motion at 1.3 m/s (3.0 mph) typically results in each foot striking the ground at a frequency of approx. 1 Hz for an adult. According to previous investigations, the dynamical vertical force can be up to 130% of the person’s body weight [3]. Assuming each foot is lifted just 1 cm from the ground, there is a potential energy of approx. 9.6 J for a 75 kg adult dissipated with every footstep considering only the vertical motion of the foot. In order to prevent the discomfort of walking on a sponge or swamp, the maximum power harvested should not be more than 10% of the foot energy. The challenge presented by this opportunity is to harvest the foot strike energy as efficiently as possible up to 100 mW (for 1 Hz walking), despite the unpredictable nature of human motion [4].

The early and most intuitive method of scavenging energy from footsteps utilized piezoelectric materials embedded in areas of footwear that experience the most strain. Shenck and Paradiso demonstrated a method of harnessing footstrike energy piezoelectrically by inserting a PZT foil into the heel area of a shoe [5]. An average power of 8.4 mW for the PZT device was reported for a foot strike frequency of 0.9 Hz. The benefit of such a design is its unobtrusiveness with respect to the user’s ergonomic experience with the product. However, the magnitude of energy that can be harnessed is limited by the use of piezoelectric devices which rely on mechanical strain created by the heel inside the shoe to create a potential difference. Moro et al. also demonstrated an energy harvesting shoe design with a system of piezoelectric vibrating cantilevers, but power generated was restricted by the low frequency of foot impact [6]. Above all, the major limitation to piezoelectric energy harvesting from walking is that the total foot energy cannot be converted to the strain of the piezoelectric material without complex additional mechanisms.

We previously developed an air pump-turbine-based system to solve the difficulties of harvesting energy from foot strikes by absorbing the foot strike energy with deformation of air bulbs and utilizing an airflow speed amplification mechanism to drive a micro-turbine [4]. The turbine was activated by airflow and operated with a high frequency, enabling the foot-strike energy to be harvested effectively. The turbine casing was designed specifically to enable the device to operate continuously with airflow from both directions [3]. A prototype was fabricated and then tested under different situations. A 6 mW peak power output was obtained with a 4.9 Ω load for each foot strike. Considering that a maximum of 100 mW could be potentially harvested as described above, there was a room for improvement.

This paper presents optimized components and system design to solve these challenges by using footsteps to compress nylon lungs embedded in a shoe sole to pneumatically drive miniature turbines and power small generators for electric power. A combat boot has been developed to provide electric power to a passive GPS tracker that can periodically ping geographical coordinates to a satellite and relay this information to a command center.

## 2. Design

**Figure 1:**
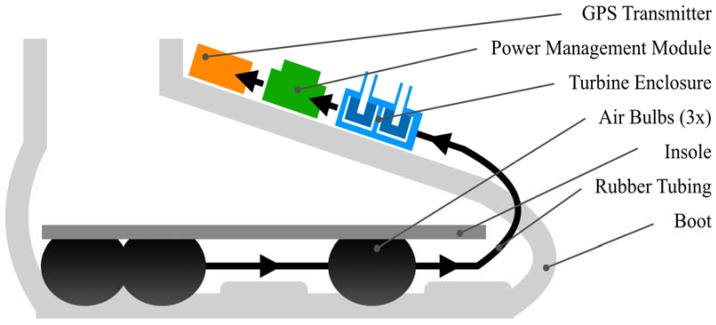
Schematic of energy harvesting system.

The system for harvesting energy presented in this paper converts able-bodied bipedal locomotion to usable electricity by way of unobtrusive hardware installed in footwear to generate airflow from the compressive forces applied by feet inside shoes during walking [7]. A diagram of this energy harvesting system embeddable in different forms of footwear is displayed in Figure 1. Air bulbs are positioned in the sole, optimized relative to the length of the foot. Outflow from the bulbs is directed towards a two-stage turbine enclosure that drives two DC motors in series to generate electrical power. This energy is stored in a supercapacitor and used to charge a battery that powers a GPS receiver.

The key design parameters considered for the air bulbs were their effective stiffness and time period of regaining original shape after being compressed. The bulbs’ effective stiffness was bound by the requirements that the bulbs not only compress under the user’s weight but also self-reset to their original decompressed form and do so fully before the user takes another step. The time available to reset depends on the frequency of foot-strikes during motion, which varies with gait and the speed of walking or running. The resultant time allowance for the air bulb to regain its initial shape can be as little as 0.3 s [8] when stepping with higher frequency. The actual air bulbs selected for testing were large nylon hand pumps used in sphygmomanometers, or blood pressure meters. The volume of each air bulb is 110 cubic centimeters, with length of 7.5 cm and diameter of 4 cm. When installed in the boot prototype, the bulbs were positioned with a tight tolerance with respect to each other and the supportive structure of the shoe interior, but still retained their shape when sitting neutrally in the shoe. Three bulbs were used for prototyping. This bulb type was only used for the proof-of-concept. Future design iterations will include embedded bulbs in the insole or outsole to minimize the discomfort to the user.

**Figure 2:**
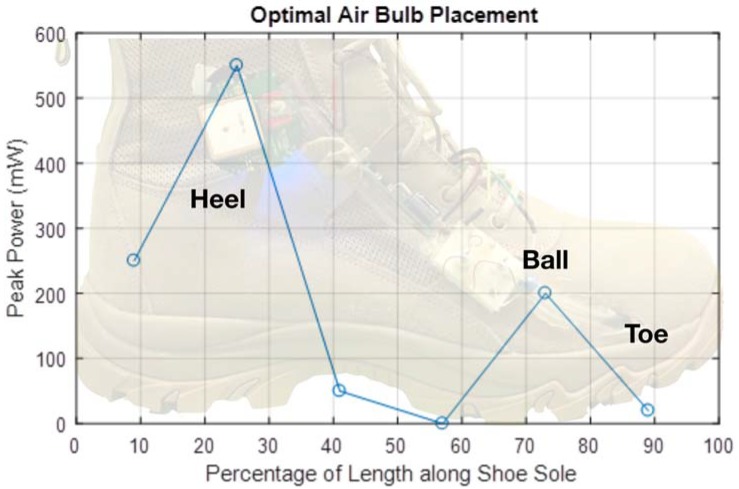
Peak power relative to length along shoe.

Optimal placement of the air bulbs along the shoe was determined by two design requirements. The first design requirement was that the chosen array of air bulbs produce the maximum air outflow with each step. The second design requirement was that the air bulbs be positioned such that the time period during which air outflow was generated was maximized during each step cycle. Furthermore, each additional air bulb added to the system increases the effective stiffness of the entire bulb array. Therefore, the design decision to add each additional airbulb to the system was based on the justification that the marginal contribution of airflow to the turbine enclosure was greater than the resultant decrease in airflow from the array when compared with the original configuration. Testing, detailed below and summarized in Figure 2, found three bulbs to be optimal given the stiffness and volume of the off-the-shelf nylon air bulbs selected for this purpose.

Positioning the air bulbs with respect to maximizing air outflow was maximized by identifying high pressure regions of the foot during walking. Although the morphology of human feet varies greatly among individuals, clinical research has shown that the pattern of weight distribution on the foot of an able-bodied human generally has maximums in the heel, ball, and toe regions of the foot [9]. In order to verify that this pattern was generally accurate for the test user of the prototype, the test user stood on a sheet of pressure indicating sensor film, which confirmed the same three regions as bearing the weight of the body.

In order to quantify how varying air bulb position along the length of the shoe directly affected energy harvesting, a single air bulb-turbine-generator assembly was developed specifically for this setup. The relative change in airflow outputted by the air bulb depending on location in the shoe was measured by observing the peak open-circuit voltage from the generator. The test setup was incrementally shifted along the length of the shoe and compressed by the weight of the human test subject at each location. The results of this test are presented in Figure 2. In order to maximize the time period of air outflow during each step cycle, spacing between groups of air bulbs was introduced to create sequential compression of the air bulbs based on the gait of human walking motion. Based on these design requirements, two air bulbs were positioned in the heel area of the sole, and one in the ball area.

**Figure 3:**
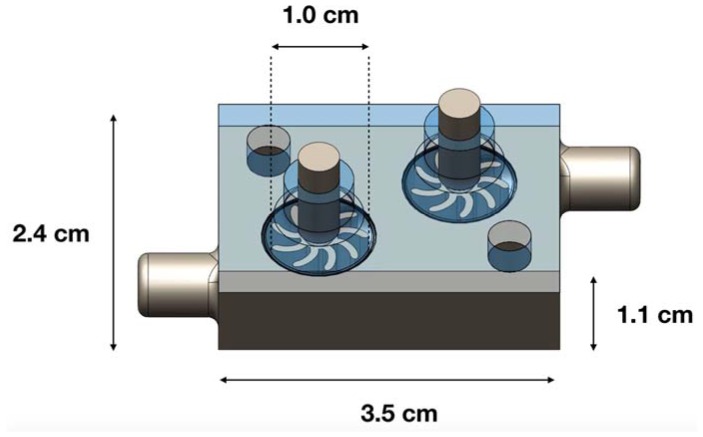
Radial symmetry of dual turbine enclosure allows for bi-directional flow to continuously drive generators.

The turbine enclosure was designed to accept flow from either the inlet (exhalation of the air bulbs during compression), or the outlet (inhalation of the bulbs during decompression), while maintaining revolution of the turbines in the same rotational direction regardless of the flow direction. Figure 3 depicts a drawing of the turbine enclosure with the top enclosure translucent to illustrate how the symmetric positioning of the two turbines allows conversion of airflow to rotational kinetic energy regardless of which inlet the air enters. The previous iteration of turbine enclosure design also geometrically enabled flow from opposite directions to rotate a single turbine in the same anti-clockwise direction, but this feature was not used to harvest energy during air bulb inhalation on the off-step. The air bulbs used in the previous iteration were off-the-shelf pumps for inflating the sleeve of a blood-pressure meter and had embedded check valves ensuring that a dedicated outlet only allowed airflow outwards. For the prototype presented in this paper, the check valves were removed, and the bulb inlet was plugged to force the air bulbs to re-inflate after compression by drawing air back through the turbine enclosure on the off-step.

The turbines themselves were designed for efficiency of airflow harvesting by optimizing blade number and internal diameter using computational fluid dynamics (CFD) [6]. The turbine blades were offset by 8.5 mm from the housing to prevent contact during use while maintaining efficiency.

The entire energy harvesting system including air bulbs, tubing, connectors, turbine components, and electronics has a mass of 130 g. Each combat boot used for testing has a mass of 770 g, so the additional weight experienced by the user is 17% of the original baseline shoe weight. Subsequent design iterations will eliminate the majority of mass from PCBs and other meso-structures when the prototype is developed for mass-application products. Since the bulbs will be embedded in cavities in the insole of the shoe, we target a net mass increase on the order of approximately 20 g.

## 3. Theoretical Calculations of Upper Bound Power

In our previous paper [3], the theoretical upper limit power by the air bulb compression and mini-turbine energy harvesting was modeled. We assumed the air bulb displacement to be 10 mm, and an amplification factor resulting from the ratio of the sectional area of the pump to that of the rubber tube inlet to be δ = 500, then the velocity of air entering the turbine can be estimated as 10 m/s for walking at 3.0 mph.

The kinetic energy for the airflow entering the turbine at relatively high velocity can be calculated from Equation (1).

(1) $$E=\frac{1}{2}m_{a}v^{2}$$

The mass of air is represented by *m_a_* and the velocity of airflow is *v*. The time period of each step was measured to be approximately 0.2 s for walking at 3.0 mph. During testing, we observed the voltage signature output with respect to time to show the working duration to be approximately 1.0 s, which includes the inhalation time after the foot step up. The calculation in Equation (1) counts only the down strike of the foot on an individual air bulb.

The energy from each step can be estimated as:

(2) $$E=\frac{1}{2}m_{a}{\left(\delta \;v_{0}\right)}^{2}=\frac{1}{2}\left(10^{-4}\times 1.29\right){\left(500\times 0.2\right)}^{2}=0.645\;J$$

Therefore, the upper limit for the power to be theoretically generated from 1.0 Hz foot strikes is approximately 645 mW. 

## 4. Prototype

A product prototype was built with the intent of applying the energy harvesting concept outlined in this paper to a real-life opportunity. The prototype presented here uses the harvested energy from walking to power a GPS module that relays geographic coordinates when requested by an SMS message from a cell phone. The motivation for selecting GPS functionality as the main application of this prototype was due to the expected use of such a product in remote environments. Potential users of energy harvesting footwear will likely be in an environment where marginal electric power can be valuable, meaning use of this product would take place far from human infrastructure capable of delivering electric power. Such users may include military personnel, emergency first-responders, and outdoor recreational explorers. 

**Figure 4:**
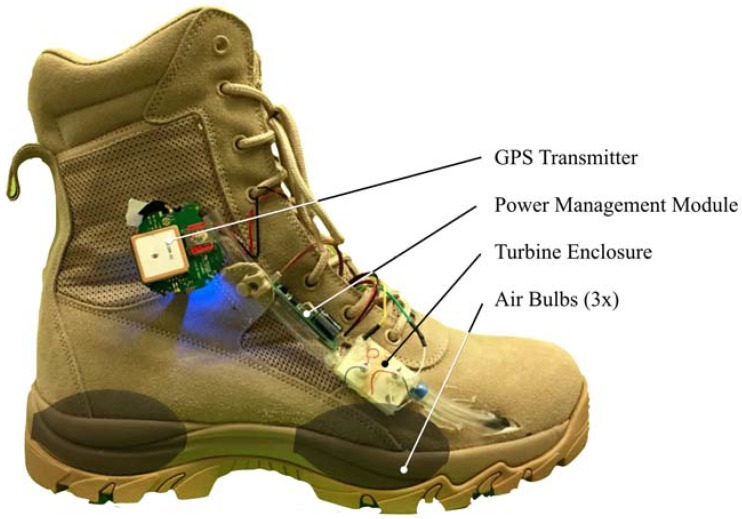
Prototype combat boot fitted with energy harvesting system.

A prototype energy harvesting system was built and installed in a combat boot, shown in Figure 4. Three air bulbs were sourced from off-the-shelf latex blood pressure measurement pumps and embedded in the shoe sole at the optimized locations. The air bulbs are transparently overlaid in Figure 4; two bulbs are positioned in the heel while one bulb is positioned in the ball region. Rubber tubing was used to direct air from the bulbs to the turbine enclosure. The turbine enclosure and the turbines themselves were 3D printed. The turbine diameter was 10 mm, with a height of 5 mm. Each turbine was directly connected to the shaft of a 1.5 V DC motor used as generators. The turbine enclosure was mounted externally to the outer side of the combat boot.

For experimentally measuring power output, the motors were connected in series to a resistor, and power was estimated by measuring potential difference across the resistor. For functional demonstration, the motors were connected to a boost converter and supercapacitor made by Advanced Linear Devices (EH4295), and the output power was regulated by a power management circuit (EH300). The supercapacitor was used to charge a Li-Po battery which powered an off-the-shelf GPS receiver. Power measurements were conducted while a 75 kg test subject walked at different controlled speeds on a treadmill while wearing the combat boot.

The off-the-shelf GPS receiver used for this prototype communicated on the 3G network. The architecture of the GPS module used for the prototype can be broken down into two functions: receiving a positioning signal from a satellite (to obtain geographic coordinates), and transmission to the GSM network (to communicate the coordinates to a cellphone). The off-the-shelf location tracker receives a low-powered microwave GPS signal from at least three or four satellites (for 2D and 3D positioning respectively). The module hardware amplifies the signal to increase gain over noise, and mixes, filters, and processes the signal in order to convert to measurements of position and time. The GPS receiver took an average of 12.6 s to communicate to the satellite, requiring 5.80 J of energy. Communicating location in the form of an SMS message to a cellphone took 13.2 s and required 6.25 J of energy. This measurement was conducted by connecting the GPS module to an oscilloscope and measuring current and voltage consumed during transmissions. The values were averaged over 8 runs.

This high power-consuming 3G GPS is actually not necessary for the basic functions provided by this prototype but was used for the ease of using off-the-shelf components for testing and demonstration in certain parts of Asia where 2G is no longer available. Using a custom-built 2G GPS unit with minimal energy needs in the future could reduce the footsteps needed per signal by an order of magnitude.

Potential strategies for reducing the power consumption of the GPS module include transmitting the geographic coordinates for longitude and latitude to fewer decimal places to reduce energy costs during transmission via GSM [10]. Additionally, separating the receiving and transmitting processes into two charging cycles to create independency between the two processes will add to the robustness of the design. An initial stage would charge a power bank and releases enough energy to power the GPS receiver and log the geolocational coordinate string for a microprocessor to store the data. The second stage would allow the stored position coordinates to be transmitted. Furthermore, increasing the efficiency of the module by customizing the digital signal processing algorithms and running the microcontroller at a lower speed would reduce the power-consumption of the hardware architecture, and allow for more frequent transmissions based on the same rate of walking.

## 5. Results

**Figure 5:**
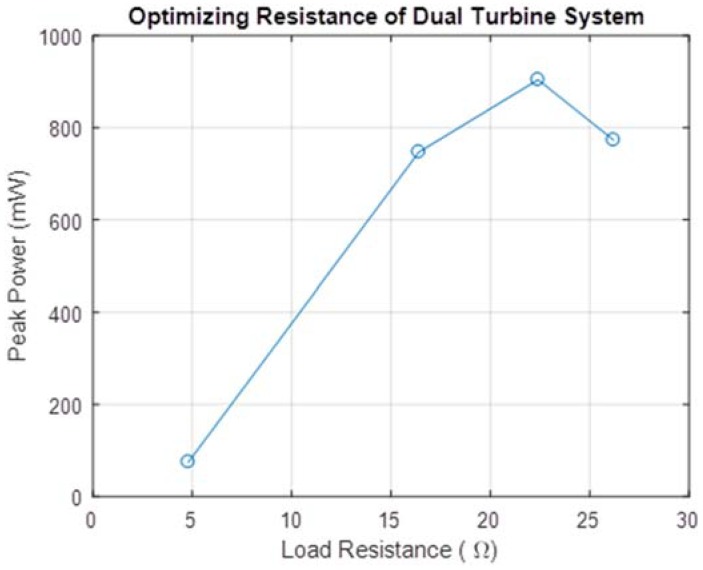
Peak power output versus values of load resistance.

The device was tested at different walking styles and speeds and voltage was measured over a 22 Ω resistor. This resistance value was optimized by measuring peak power output for a footstep cycle for various load resistances, shown in Figure 5.

**Figure 6:**
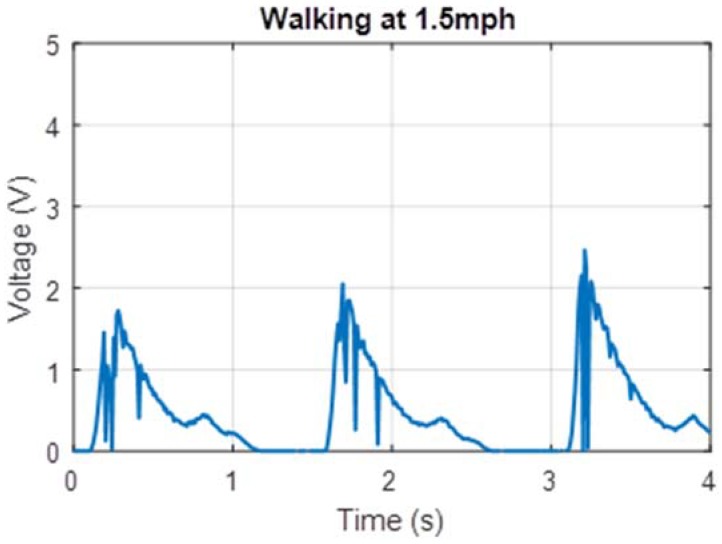
Voltage output measured for 0.5 Hz footsteps.

**Figure 7:**
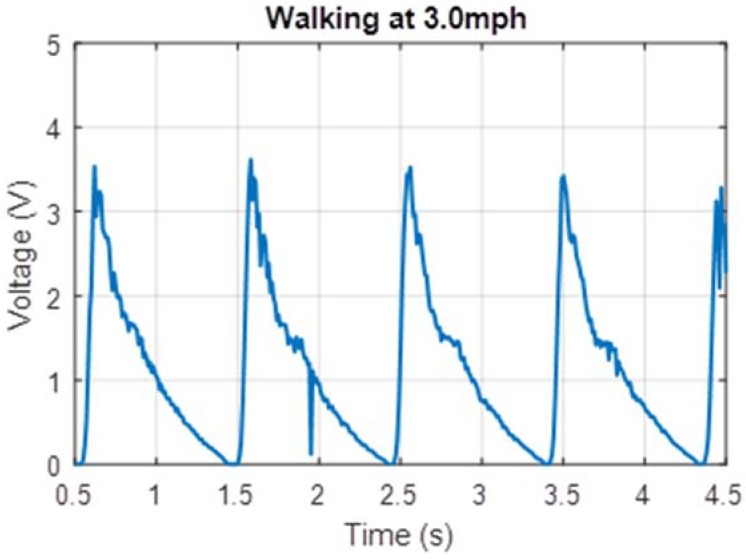
Voltage output measured for 1.0 Hz footsteps.

**Figure 8:**
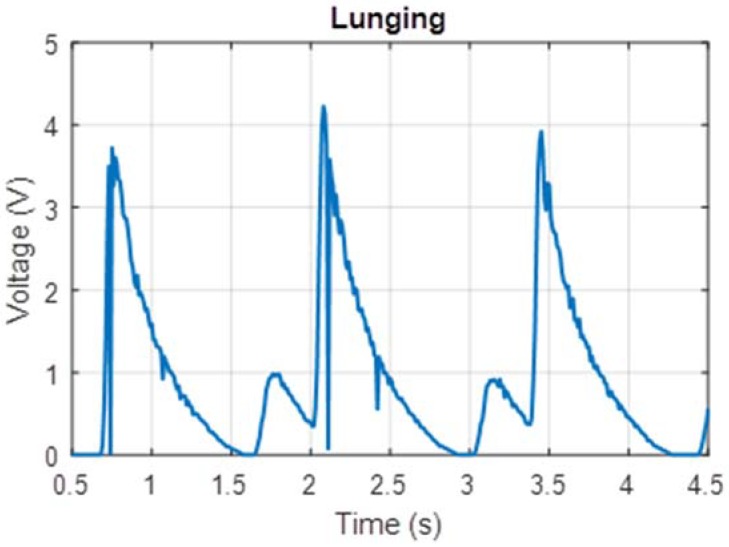
Voltage output measured for Lunges.

The human factor involved with harvesting energy from footsteps meant that such a device would naturally be subjected to a variety of types of movement by users with varying weights. Due to experimental constraints, a single user of weight 75 kg tested the prototype. The prototype was tested on a treadmill at two different walking speeds (1.5 mph and 3.0 mph) which corresponded to foot strikes at frequencies of 0.5 Hz and 1.0 Hz respectively. Voltage measured across a 22 Ω resistor for these cases is shown in Figure 6 and Figure 7. Additionally, power measurements were taken while the test subject lunged, attempting to simulate maximum impact to the shoe. These voltage measurements are shown in Figure 8.

The power measurements in Figure 6, Figure 7 and Figure 8 are based on measurements for voltage over the known resistor load and estimated using the equation expressed in Equation (3) relating electric power *P* to potential difference *V* and load resistance *R*.

(3) $$P=\frac{V^{2}}{R}$$

The peak power for these tests can be estimated by relating peak voltage and known load resistance, 22 Ω in these cases. The average continuous power was found by calculating the total electrical energy generated over a six-second interval of use and dividing by time. These values are displayed in Table 1.

While power measurements were conducted while the test subject moved in controlled motions on a treadmill, the functional demonstration of the GPS capabilities of the prototype was conducted in a remote area on uneven terrain, allowing the user to create random motions similar to those experienced during routine use. The off-the-shelf GPS receiver used for testing required 15 min of activity in the combat boot before enough energy was produced to send a text message with geographical coordinates. 

From Equation (2), the upper theoretical limit for power output by the turbine was 645 mW at footstep frequency of 1.0 Hz. Based on the average power measurement in Table 1, this prototype has achieved capturing 13.4% of the foot strike energy. This value is comparable to the initial goal of capturing 10% of the foot strike energy as a goal to balance maximizing harvested energy and minimizing side effects on the user’s experience while wearing the shoe during use.

These power measurements in Figure 6, Figure 7 and Figure 8 are based on measurements for voltage over a known resistor load and estimated using Equation (3). During measurement, these were not connected to the voltage booster or the capacitor module. During prototype testing, the charging voltage (per cycle) provided to the voltage booster was approximately an average 0.5 V at 0.5 Hz, and the subsequent charging voltage to the capacitor in the power management module was clamped at 12 V. Based on the off-the-shelf PCB that was used, the total time for a full transmission (gathering data from GPS receiver, processing the data and sending coordinates information via the 3G local network) was less than 70 s. The total energy required for the entire transmission is approximately 31.6 J. Based on a simplified RC circuit and understanding that the EH300 module useful energy output operates between 3.5 V to 1.9 V, the capacitance required to power the PCB such that we can send a single transmission is approximately 22 farads. Constrained by the energy requirements of the load, the off-the-shelf PCB is not practical for passive use with the proposed electronics hardware. Instead a trickle charge circuit can be used to extend the life of a battery that operates this particular GPS module. As this is an off-the-shelf module, there leaves little opportunities for customization and optimization to a low-power circuit.

Nonetheless, removing additional functionalities such as Bluetooth and constant location tracking in the off-the-shelf PCB, it is possible to achieve the aim of a self-sustainable tracking circuit. Using low powered GPS receivers and microcontrollers, and operating on the 2G network, a low-powered circuit may be achieved. Low powered GPS receivers have a fast time to first fix (TTFF) of approximately 26 s from cold start and power consumption of less than 20 mW. The estimated operating time for such a circuit is approximately 41 s and requires a total energy of 2.1 J. A suitable supercapacitor rating of 2.1 farads can be used for an energy input from 15 min of walking at 0.5 Hz.

Other than using energy efficient hardware and peripheral functions, optimization to the algorithm has an impact on energy consumption as well. For example, the off-the-shelf PCB transmits a string of data containing excess information including a link to Google Maps, full date and time, speed of the tracker, battery level and tracker identification number. A total of 136 characters is used and an approximate energy consumption of 2.2 J. Using a 2G network and transmitting just the necessary coordinates yields about 1.5 J [10].

Other ways to ease the energy consumption of the tracker circuit includes reducing the TTFF by adopting efficient correlation algorithms and optimizing antenna design. The EH300 module also allows recharging on the “on” state with a voltage clamp of 7 V. Thus, the low-power circuit can be sustained longer than 41 s as long as the user keeps walking.

## 6. Discussion

This paper presents an energy harvesting system designed to convert footsteps into usable electric power. The device used an array of air bulbs that sequentially compressed and decompressed during the user’s walking motion, to continuously drive a series of miniature turbines to generate energy. The turbine enclosure was designed specifically to accept flow from opposite directions while ensuring the turbines themselves always turned in the same rotational direction. As a result, at a moderate walking speed of 3.0 mph (footstep frequency of approximately 1 Hz), there was no measurable down-time when the turbines were not rotating and producing electric power.

Because the energy harvesting device depends on human movement, it is difficult to quantify its performance with respect to a wide range of users. The prototype presented in this paper was tested on a 75 kg subject and generated an average continuous power between 30 mW and 80 mW depending on the gait and speed of the subject’s movement. Approximately 15 min of varied forms of movement was required to generate enough energy to send a GPS transmission detailing geographical coordinates, sent as a text message to a cell phone. 

If the airflow generation and air bulb compression is further optimized within the sole of the boot, the power output can be improved to the theoretical prediction of the previous section. Considering the GPS receiver used in this prototype required very high power for transmission to satellites, this combat boot can be optimized to provide more power at each step to a lower power consuming GPS in order to deliver transmissions more frequently.

Future work on this device will involve optimizing the circuit design by developing a custom designed power management system and low-power GPS receiver to reduce the number of footsteps required to operate as well as to improve the performance of the air bulb-turbine efficiency. In addition, ergonomic factors not considered in this design, such as the effect of prolonged, repetitive use on the user’s feet, need to be taken into account and incorporated into the final product-oriented design.

## Figures and Tables

**Table 1:** Power generated for various modes of movement.

Movement Type	Average Power (mW)	Peak Power (mW)
0.5 Hz Footsteps	29.9	161
1.0 Hz Footsteps	86.4	516
Lunges	106	679
